# Methylglyoxal-Derived Advanced Glycation Endproducts Accumulate in Multiple Sclerosis Lesions

**DOI:** 10.3389/fimmu.2019.00855

**Published:** 2019-04-24

**Authors:** Suzan Wetzels, Tim Vanmierlo, Jean L. J. M. Scheijen, Jack van Horssen, Sandra Amor, Veerle Somers, Casper G. Schalkwijk, Jerome J. A. Hendriks, Kristiaan Wouters

**Affiliations:** ^1^Cardiovascular Research Institute Maastricht, Department of Internal Medicine, Maastricht University, Maastricht, Netherlands; ^2^Biomedical Research Institute, Department of Immunology and Biochemistry, Hasselt University, Hasselt, Belgium; ^3^Department of Psychiatry and Neuropsychology, School for Mental Health and Neuroscience, Maastricht University, Maastricht, Netherlands; ^4^Department of Molecular Cell Biology and Immunology, Amsterdam UMC, Vrije Universiteit, Amsterdam, Netherlands; ^5^Department of Pathology, Amsterdam UMC, VU University Medical Center, Amsterdam, Netherlands

**Keywords:** multiple sclerosis, neuroinflammation, α-dicarbonyl, advanced glycation endproducts, astrocytes

## Abstract

Multiple sclerosis (MS) is a demyelinating autoimmune disease in which innate and adaptive immune cells infiltrate the central nervous system (CNS) and damage the myelin sheaths surrounding the axons. Upon activation, infiltrated macrophages, CNS-resident microglia, and astrocytes switch their metabolism toward glycolysis, resulting in the formation of α-dicarbonyls, such as methylglyoxal (MGO) and glyoxal (GO). These potent glycating agents lead to the formation of advanced glycation endproducts (AGEs) after reaction with amino acids. We hypothesize that AGE levels are increased in MS lesions due to the inflammatory activation of macrophages and astrocytes. First, we measured tissue levels of AGEs in brain samples of MS patients and controls. Analysis of MS patient and non-demented control (NDC) specimens showed a significant increase in protein-bound N^δ^-(5-hydro-5-methyl-4-imidazolon-2-yl)-ornithine (MG-H1), the major AGE, compared to white matter of NDCs (107 ± 11 vs. 154 ± 21, *p* < 0.05). In addition, immunohistochemistry revealed that MGO-derived AGEs were specifically present in astrocytes, whereas the receptor for AGEs, RAGE, was detected on microglia/macrophages. Moreover, in cerebrospinal fluid from MS patients, α-dicarbonyls and free AGEs correlated with their respective levels in the plasma, whereas this was not observed for protein-bound AGEs. Taken together, our data show that MG-H1 is produced by astrocytes. This suggests that AGEs secreted by astrocytes have paracrine effects on RAGE-positive macrophages/microglia and thereby contribute to the pathology of MS.

## Introduction

Multiple sclerosis (MS) is an inflammatory disease of the central nervous system (CNS) and is the major cause of disability in young adults in Western countries (1). Although the exact trigger of MS remains unidentified, an autoimmune response against the myelin sheaths is widely-considered involved in disease onset and progression. This autoimmune response is caused by an interplay between the innate immune system and the adaptive immune system (2). Autoreactive T-lymphocytes are recruited to the CNS and reactivated by myelin phagocytosing macrophages and microglia, thereby promoting neuroinflammation and neurodegeneration (3). In addition to immune cells, CNS-resident astrocytes contribute to the neuroinflammatory response by secreting pro-inflammatory cytokines and chemokines (4). Most patients (85%) present with the relapsing-remitting MS disease course in which relapses result in episodes of disability with full recovery between the relapses (1). Over half of these patients enter the secondary progressive phase after 5–15 year of diagnosis which is characterized by progression of the disease without full recovery caused by axonal damage (5).

The pro-inflammatory activation of immune and glial cells such as macrophages, CNS-resident microglia and astrocytes induces a switch in metabolism favoring glycolysis (6–8). During glycolysis, methylglyoxal (MGO) is produced from glyceraldehyde-3-phosphate and dihydroxyacetone phosphate, and glyoxal (GO) directly from glucose (9, 10). MGO and GO are major precursors in the formation of advanced glycation endproducts (AGEs) (10). The interaction of MGO with arginine leads to the formation of methylglyoxal-derived N^δ^-(5-hydro-5-methyl-4-imidazolon-2-yl)-ornithine (MG-H1), whereas its interaction with lysine leads to the formation of N^ε^-(1-carboxyethyl)-lysine (CEL) (11). Furthermore, interaction of GO with lysine leads to the formation of N^ε^-(carboxymethyl)-lysine (CML) (11). Degradation of protein-bound AGEs results in single modified amino acids. AGEs, both in their free and protein-bound form, are able to bind the surface receptor for advanced glycation endproducts (RAGE). This leads to the activation of downstream signaling pathways inducing oxidative stress and NF-κB activation, which in turn results in the production of pro-inflammatory cytokines (12, 13). To protect our body against increased levels of MGO and GO, the glyoxalase system, consisting of glyoxalase 1 (GLO1) and glyoxalase 2 (GLO2), degrades MGO, and GO (14), thereby limiting RAGE activation and subsequent inflammation.

Previous research shows that AGEs accumulate in the adipose tissue during obesity (15), in atherosclerotic plaques (16, 17) and in the retina during diabetes (16, 18), diseases which are all characterized by inflammation. AGEs also accumulate in the brain during neurodegenerative and neuroinflammatory diseases such as in Parkinson's patients (19), and in the cerebrospinal fluid (CSF) of Alzheimer's patients (20). Furthermore, Sternberg and colleagues have revealed that AGEs and RAGE are present in the hippocampus of MS patients (21). They also found that plasma protein-bound CEL levels are increased in MS patients (22). More recently, we have revealed that AGEs are increased in the spinal cord of mice subjected to experimental autoimmune encephalomyelitis, an inflammatory animal model of MS (23). Based on the above findings, we hypothesize that AGE levels are increased in MS lesions due to the inflammatory activation of macrophages and astrocytes. In this study, we determined the cellular distribution and quantitated tissue levels of AGEs in brain samples of MS patients. In addition, we determined whether the levels of α-dicarbonyls and AGEs in the CSF correlate with plasma and elucidated whether these levels correlate with disease parameters.

## Materials and Methods

### Sample Collection CSF Study Population

CSF and paired plasma samples of MS patients (*n* = 18) were obtained from the University Biobank Limburg, Belgium. The group of MS patients consist of 9 relapsing remitting MS, 8 secondary progressive MS and 1 clinically isolated syndrome patient. Medication use included the use of Tysabri® (*n* = 1), Gilenya® combined with Methotrexate® (*n* = 1), Endoxan® (*n* = 1), Copaxone® (*n* = 1), and Methotrexate® (*n* = 2). Twelve MS patients were untreated. The study protocol was approved by the Medical Ethical committee of the Jessa Hospital and of Hasselt University, Hasselt, Belgium.

### Sample Collection and Preparation of MS Lesions

Frozen post-mortem tissue blocks containing brain lesions (*n* = 15) of MS patients and white matter (*n* = 10) of non-demented controls (NDCs) were obtained from the Netherlands Brain Bank. The samples were matched for age and gender. MS lesions were characterized by the Netherlands Brain Bank as active, chronic active and chronic inactive (*n* = 5/group). Patients diagnosed with type I or II diabetes mellitus were excluded from this study. The study protocol was approved by the Medical Ethical committee of Hasselt University and of the Jessa Hospital, Hasselt, Belgium.

The post-mortem brain tissues containing brain lesions of MS patients and white matter of NDCs were homogenized in 0.1 M sodium phosphate buffer, pH 6.8, containing 0.02% Triton-X (VWR International, Radnor, USA) and protease inhibitor cocktail (Roche, Basel, Switzerland). Tissue lysates were used to measure α-dicarbonyls, AGEs and GLO1 enzyme activity.

### α-Dicarbonyl and AGE Measurements

α-Dicarbonyls methylglyoxal (MGO), glyoxal (GO), and 3-deoxyglucosone (3DG), and AGEs N^ε^-(carboxymethyl)lysine (CML), N^ε^-(1-carboxyethyl)lysine (CEL), and N^δ^-(5-hydro-5-methyl-4-imidazolon-2-yl)-ornithine (MG-H1) were analyzed in the plasma, CSF, and post-mortem tissue samples using ultra-performance liquid chromatography tandem mass spectrometry. Protein bound AGEs were corrected for the amount of lysine, determined with the same measurement and in the same samples, as a measure for the amount of total protein, as described previously (24, 25).

### Immunohistochemistry

Paraffin sections of MS lesions and white matter of NDCs were sectioned 7 μm thick using a Leica microtome (Leica, Wetzlar, Germany). Sections were deparaffinised using xylene and rehydrated following a decreasing ethanol range. Incubation for 10 min at 37°C with citrate buffer (1.6 mM citric acid and 8.4 mM trisodium citrate, pH 6.0) was used as antigen retrieval method. Thereafter, sections were blocked using 1% BSA (Sigma-Aldrich, Saint Louis, USA) in phosphate buffered saline (PBS). Anti-MGO-derived AGEs [1:12.5 custom made, 1:84 biotin labeled, custom made (16)], anti-GFAP (1:500, Sigma-Aldrich, Saint Louis, USA), anti-Iba1 (1:500, Wako Chemicals, Neuss, Germany), and anti-neurofilament (1:750, Sigma-Aldrich, Saint Louis, USA) were used as primary antibodies. After washing with PBS, sections were incubated for 1 h with the appropriate FITC and TRITC fluorescently labeled secondary antibodies (1:600, Invitrogen, Carlsbad, USA). DAPI staining, 10 min at room temperature, was used to visualize cell nuclei. Sections were photographed using Leica fluorescent microscope (Leica, Wetzlar, Germany) at 40x magnification.

Frozen MS lesions were cryosectioned at 5 μm thickness. Sections were air-dried for 30 min, fixed for 10 min in acetone and subsequently washed in PBS. Anti-RAGE (1:50, Santa Cruz Biotechnology, Dallas, USA), anti-Iba1 (1:500, Wako Chemicals, Neuss, Germany), and anti-GFAP (1:300, Dako-Agilent, Santa Clara, USA) were used as primary antibodies. After washing with PBS, sections were incubated for 1 h with the appropriate FITC and TRITC fluorescent labeled secondary antibodies (1:600, Invitrogen, Carlsbad, USA). DAPI was used to stain cell nuclei. Sections were photographed using Leica fluorescent microscope (Leica, Wetzlar, Germany) at 40x magnification.

### GLO1 Activity Assay

GLO1 activity was measured in protein lysates of human tissue as described previously by McLellan et al. (26). In short, GLO1 activity was measured using a spectrophotometry analysis by determining the formation of S-D-Lactoylglutathione from MGO at an absorbance of 240 nm during 30 min.

### Statistical Analysis

Statistical analysis were performed using SPSS Statistics software, version 24 (IBM Corporation, Armonk, USA) or GraphPad Prism version 7 (GraphPad Software, La Jolla, USA). Baseline characteristics of post-mortem samples from NDCs and MS patients were analyzed using one-way ANOVA with Tukey's multiple comparisons test (GraphPad Prism). α-dicarbonyl and AGE levels of the post-mortem material were analyzed using one-sided unpaired *t*-test (GraphPad Prism), based on our previous results obtained from our mouse model (23). Partial correlation analysis was used to determine CSF α-dicarbonyls and AGEs and disease parameters expanded disability scale score (EDSS), number of relapses and disease duration. These data were corrected for age, gender, medication use and glucose concentration in the CSF using SPSS statistics. Linear regression analysis was used to determine associations between plasma α-dicarbonyls and AGEs and CSF α-dicarbonyls and AGEs (GraphPad Prism). All data are presented as mean ± SEM. *P* ≤ 0.05 was considered statistically significant.

## Results

### MG-H1 Is Increased in the Lesion Area of MS Patients

**Figure 1:**
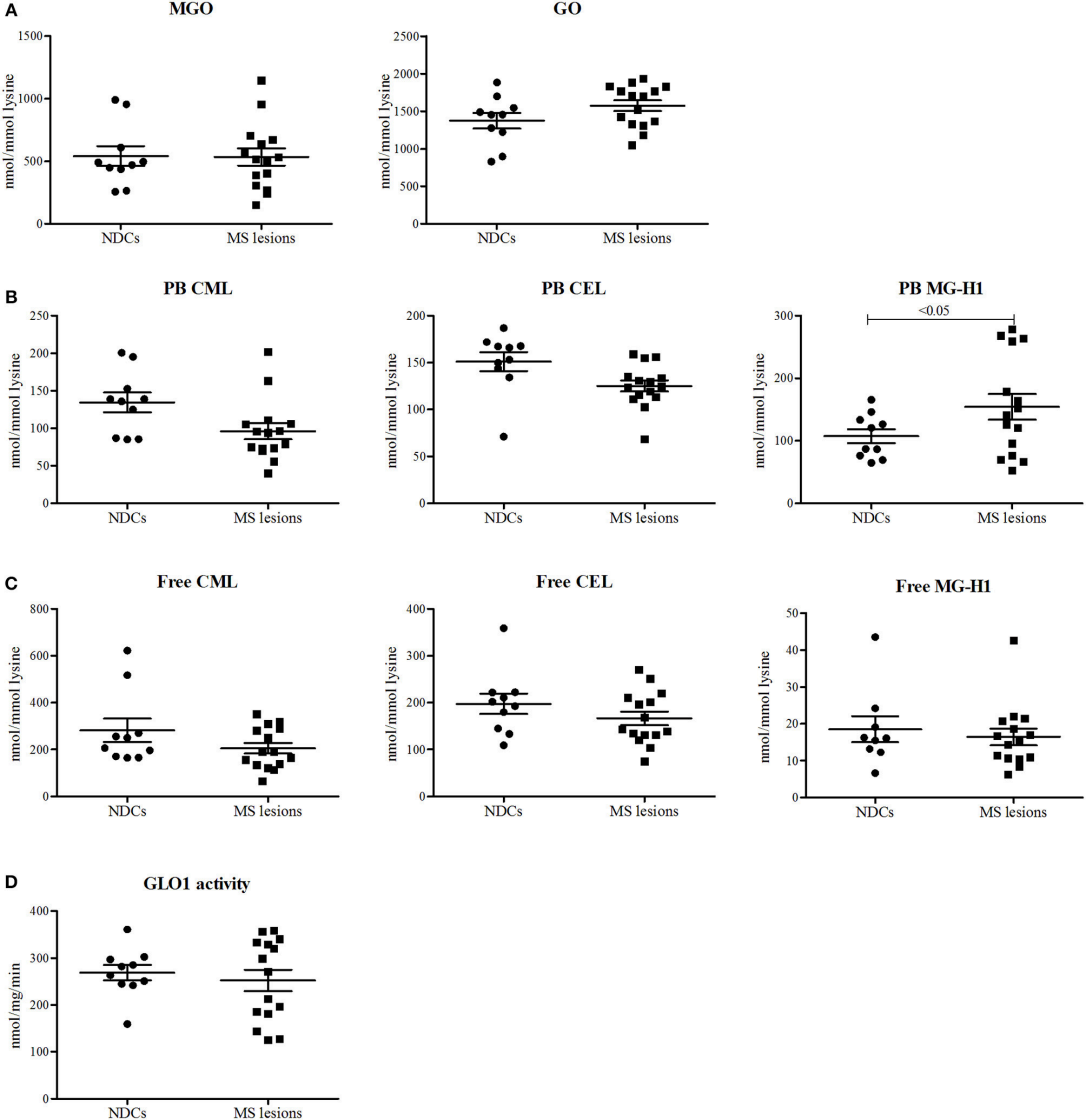
Protein-bound MG-H1 is increased in post-mortem MS lesions compared to white matter of NDCs. α-dicarbonyls MGO and GO **(A)**, protein-bound (PB) CML, CEL, and MG-H1 **(B)**, free CML, CEL, and MG-H1 **(C)**, and GLO1 activity **(D)** was determined in white matter post-mortem samples obtained from non-demented controls (NDCs), and in post-mortem samples of MS lesions. Data is presented as Mean ± SEM. Data is analyzed using one-sided unpaired *t*-test.

To determine whether the levels of α-dicarbonyls and AGEs are increased in the lesions of MS patients compared to white matter of NDCs, post-mortem lesions of 15 MS patients and 10 white matter samples of NDCs were obtained (Table 1) and levels of MGO, GO and free and protein-bound CML, CEL, and MG-H1 were measured. The MS lesions were subdivided into three categories: active lesions, chronic active lesions, and chronic inactive lesions. Post-mortem delay (until storage) was significantly higher for the chronic inactive lesions (Table 1). However, there was no correlation between post-mortem delay and α-dicarbonyl, protein-bound, and free AGE levels, arguing that post-mortem delay did not affect the measurements (Supplemental Figure 1). Levels of MGO and GO were not altered in MS lesions compared to white matter of NDCs (Figure 1A). Interestingly, protein-bound MG-H1, the major MGO-derived AGE, was significantly higher in MS lesions (*p* < 0.05), whereas CML and CEL were not different (Figure 1B). Free AGE levels did not differ between MS lesions and brain tissue of NDCs (Figure 1C). GLO1 activity was unaltered in MS lesions compared to controls (Figure 1D). Together, these data demonstrate that MG-H1 accumulates in the lesions of MS patients, irrespective of the activity of GLO1.

**Table 1:** Baseline characteristics of post-mortem material of MS patients (*n* = 15) and NDCs (*n* = 10).

	**Active MS lesion(*n =* 5)**	**Chronic active MS lesion(*n =* 5)**	**Chronic inactive MS lesion(*n =* 5)**	**NDCs (*n =* 10)**
Age	69 ± 5	65.4 ± 7	58.8 ± 4	71.1 ± 0.8
Female, %	40%	60%	40%	70%
Post-mortem delay (minutes)	549 ± 36	543 ± 47	606 ± 39^*^	427 ± 39

* *p < 0.05 compared to NDCs*.

*Data is presented as mean ± SEM and analyzed using one-way ANOVA and Tukey's multiple comparisons test*.

### MGO-Derived AGEs Are Present in Astrocytes in MS Lesions

**Figure 2:**
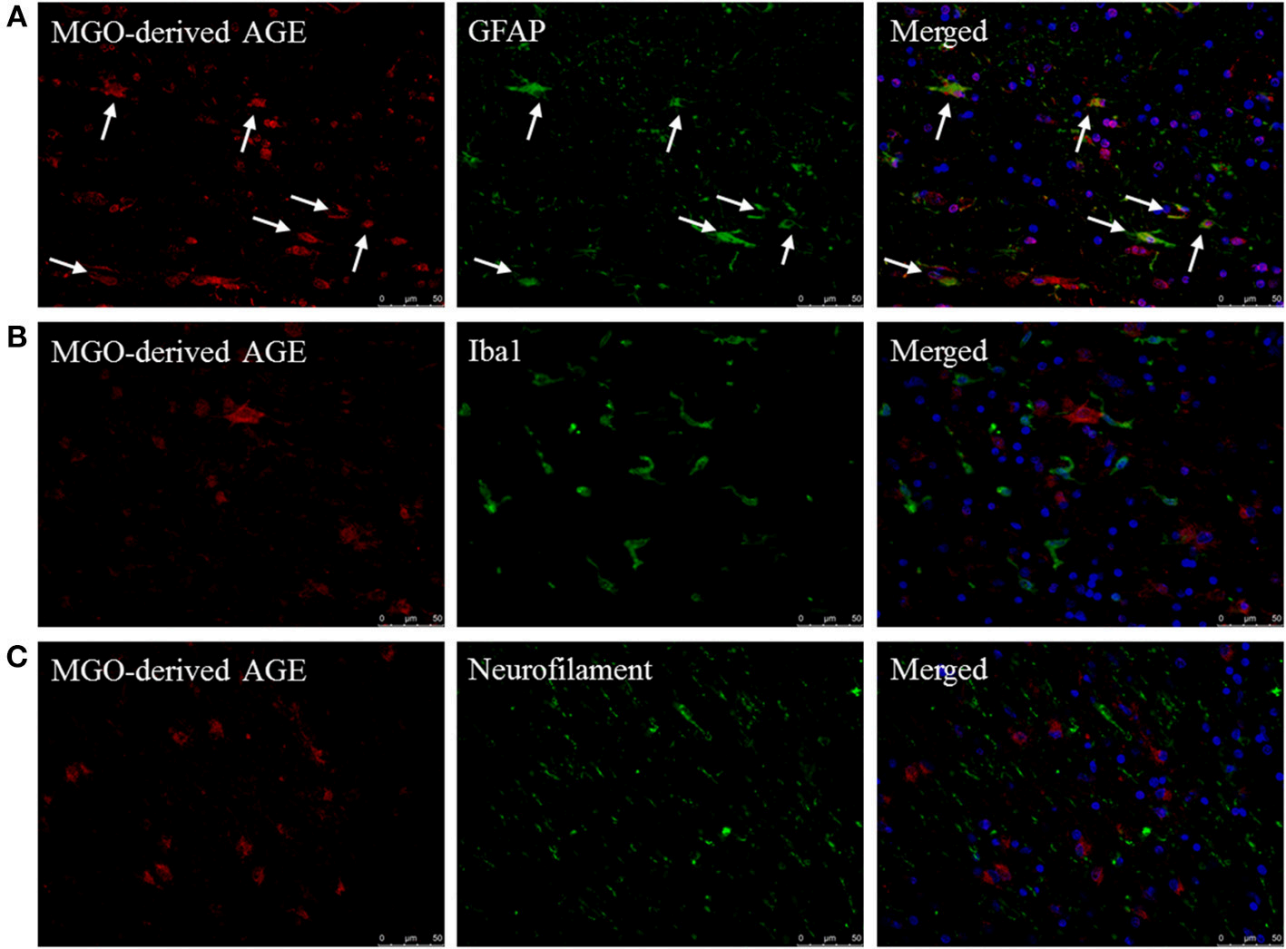
MGO-derived AGE accumulates predominantly in astrocytes. Staining of MGO-derived AGE (red, TRITC) combined with GFAP (green, FITC) **(A)**, Iba1 (green, FITC) **(B)**, and neurofilament (FITC) **(C)** show that MGO-derived AGE accumulates in astrocytes in normal appearing white matter and lesions of MS patients as indicated by the white arrows. Nuclei were stained with DAPI (blue). Representative of *n* = 4 staining.

To determine which cell type mainly contributes to AGE production in the CNS, fluorescent double staining was performed on MS lesions to localize MGO-derived AGEs combined with cell makers for astrocytes (GFAP), macrophages/microglia (Iba1), and neurons (neurofilament). MGO-derived AGE was detected in sections that contained both lesion and NAWM. These stainings revealed that MGO-derived AGEs are present in MS lesions and normal appearing white matter of MS patients and primarily co-localize with GFAP^+^ astrocytes (Figure 2A, indicated by the white arrows). MGO-derived AGEs did not show co-localization with Iba1^+^ macrophages/microglia (Figure 2B) or with neurofilament^+^ neurons (Figure 2C). In addition to MS lesions and NAWM, we performed these stainings in white matter of NDCs and confirmed that MGO-derived AGE was only present in the GFAP^+^ astrocytes (Supplemental Figure 2).

**Figure 3:**
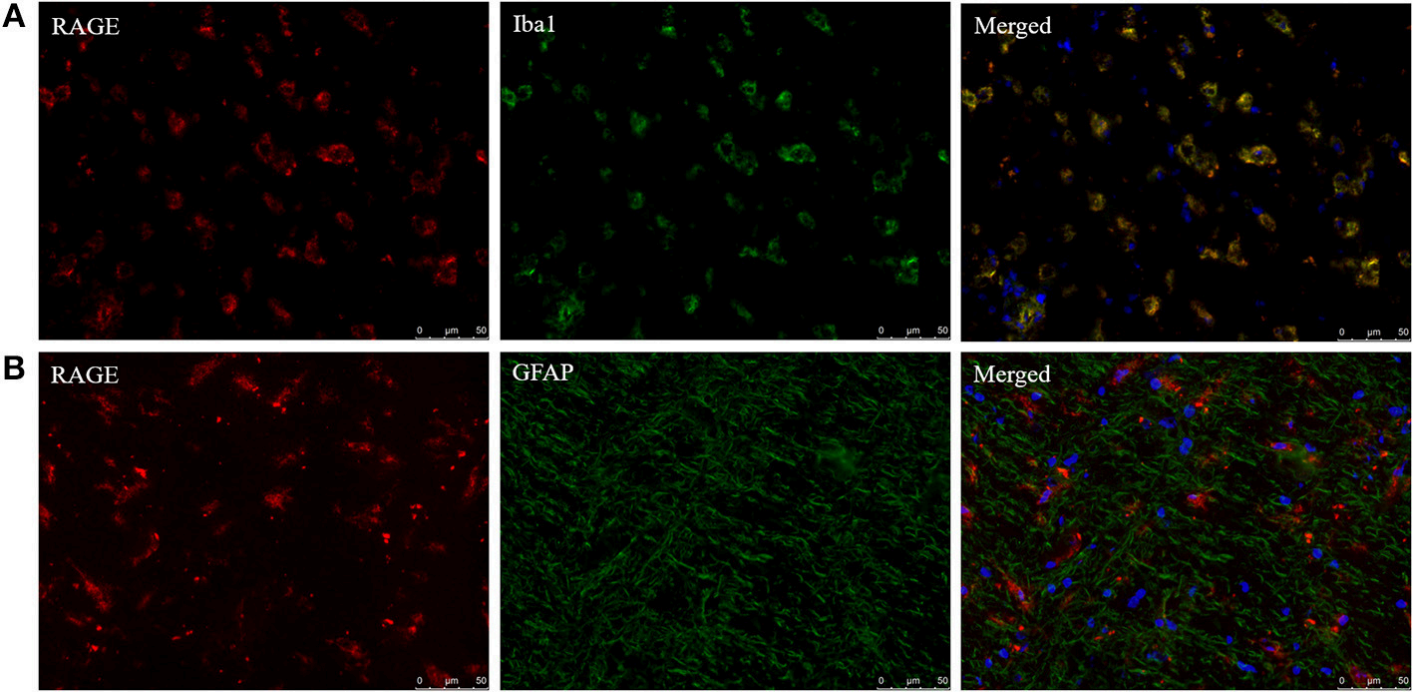
Iba1^+^ cells express the receptor of AGEs. Staining of RAGE (red, TRITC) combined with Iba1 (green, FITC) **(A)** and GFAP (green, FITC) **(B)** show that RAGE is present on Iba1^+^ macrophages/microglia and absent on GFAP^+^ astrocytes in the normal appearing white matter and lesions of MS patients. Nuclei were stained with DAPI (blue). Representative of *n* = 4 staining.

In addition to AGE localization, RAGE distribution was examined with fluorescent double staining to identify the cell types capable of responding to AGE formation in the CNS. RAGE was detected in sections that obtained both MS lesion and NAWM. RAGE expression co-localized with Iba1^+^ macrophages/microglia (Figure 3A) while GFAP^+^ astrocytes were not positive for RAGE (Figure 3B). Moreover, we observed no presence of RAGE on neurons using DAB-staining (data not shown).

### α-Dicarbonyls and AGEs in CSF Do Not Correlate With Disease Parameters of MS

To assess whether α-dicarbonyl and AGE levels in the CSF are correlated with disease parameters of MS, partial correlation analysis was performed. CSF samples of MS patients were obtained from the University Biobank Limburg. Baseline characteristics of the study population are shown in Table 2. No correlation was found between CSF AGE levels and EDSS, the number of relapses, and duration of the disease (Table 3). Interestingly, a significant negative correlation was found between free MG-H1 in CSF and disease duration (*p* < 0.05), and between protein-bound MG-H1 and EDSS (*p* < 0.05) (Table 3).

**Table 2:** Baseline characteristics from MS patients (*n* = 18) included in the CSF study population.

	**MS patients (*n =* 18)**
Age	43.8 ± 3
Gender (female), %	78%
[glucose] _CSF_, mg/dL	61.3 ± 1
Medication use, %	33%
Clinically isolated syndrome, %	6%
Relapsing remitting MS, %	50%
Secondary progressive MS, %	44%
EDSS	4.2 ± 0.6
Duration of disease, years	6.4 ± 2
Number of relapses	2.6 ± 0.3

*Duration of disease is determined as time between diagnosis and sampling of CSF. Data is presented as Mean ± SEM or as percentage of the total group*.

**Table 3:** Correlation of CSF α-dicarbonyl and AGE levels with MS disease parameters.

	**Correlations (*****R***^****2****^**)**		
	**EDSS**	**Number of relapses**	**Disease duration, years**
MGO	−0.175	−0.023	−0.153
GO	−0.378	0.213	0.008
3-DG	−0.242	−0.062	0.082
Free CML	0.125	−0.209	−0.357
Free CEL	−0.32	−0.134	−0.165
Free MG-H1	0.284	0.014	−0.542^*^
PB CML	−0.18	0.377	−0.152
PB CEL	−0.099	0.306	−0.452
PB MG-H1	−0.551^*^	0.029	−0.12

* *p < 0.05. Correlation is determined between MGO, GO, and 3DG, protein-bound (PB) CML, CEL, and MG-H1, and free CML, CEL, and MG-H1 in the CSF with EDSS, number of relapses and disease duration. Data is analyzed using partial correlation analysis and corrected for age, gender, medication use, and [glucose]_CSF_*.

### α-Dicarbonyls and Free AGEs in CSF Correlate With Plasma Levels

**Figure 4:**
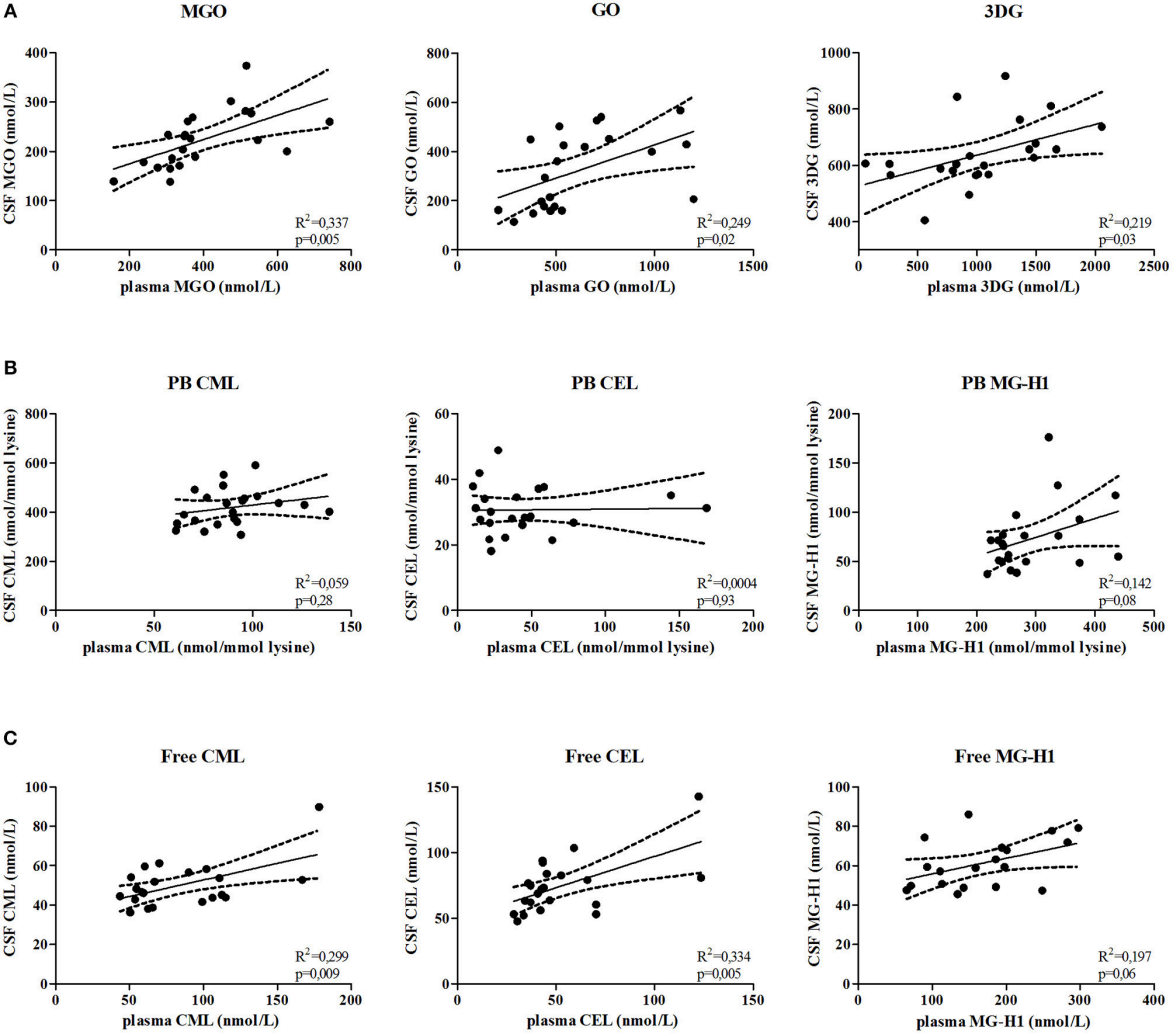
α-dicarbonyls and free AGEs in the plasma and CSF are correlated. Correlation is determined between MGO, GO, and 3DG **(A)**, protein-bound (PB) CML, CEL, and MG-H1 **(B)** and free CML, CEL, and MG-H1 **(C)** in the CSF and in the plasma. Data is analyzed using linear regression analysis and shows regression line with 95% confidence band.

To determine whether AGE levels in plasma and CSF correlate, α-dicarbonyl, free AGEs, and protein-bound AGEs were determined in paired plasma and CSF samples of MS patients. Plasma MGO (*p* = 0.005), GO (*p* = 0.02), and 3DG (*p* = 0.03) levels significantly correlated with CSF levels (Figure 4A). In addition, the free AGEs levels of CML (*p* = 0.009), and CEL (*p* = 0.005) in plasma significantly correlated with their CSF counterparts, and plasma levels of MG-H1 (*p* = 0.06) tended to be correlated with CSF levels (Figure 4B). However, plasma protein-bound CML, CEL and MG-H1 did not correlate with CSF levels (Figure 4C).

## Discussion

In this study we showed that MGO-derived MG-H1 is significantly increased in MS lesions and is mostly present in astrocytes. In addition, we revealed that α-dicarbonyl and AGE levels in the CSF do not correlate with disease parameters, but do correlate with plasma levels.

AGE levels were measured in post-mortem samples of NDCs and MS patients. This analysis revealed that protein-bound MG-H1 levels were increased in MS lesions compared to the levels in the white matter of NDCs. Protein-bound CML and CEL levels and free AGE levels were comparable between MS lesions and white matter of NDCs. Since MG-H1 is the major MGO-derived AGE (11), the higher levels of MG-H1 suggest enhanced MGO production in the lesions of MS patients. However, MGO levels were not different in MS lesions compared to NDCs. Possibly, the highly reactive MGO that is formed intracellularly rapidly interacts with cellular proteins to form protein-bound AGEs. In addition, excessively formed MGO may also rapidly leave the cell due to simple diffusion given its small molecular weight. In addition, we and others have previously shown that inflammation decreases GLO1 activity (17, 23). The activity of GLO1, the major MGO detoxifying enzyme, was similar between the white matter of NDCs and MS lesions. These results indicate that the increased levels of MG-H1 are likely due to increased MGO formation and not due to decreased degradation by GLO1.

We found that MGO-derived AGEs mainly accumulate in GFAP^+^ astrocytes in human MS lesions and in white matter of NDCs. Moreover, in accordance with the findings of Barateiro et al. (27), double staining of GFAP and Iba1 with RAGE showed that RAGE was expressed on Iba1^+^ microglia/macrophages in MS lesions, and not on GFAP^+^ astrocytes. These data indicate that AGEs are produced in activated glycolytic astrocytes and could exert paracrine effects by binding RAGE on microglia/macrophages. Activation of RAGE results in the activation of NF-κB, which in turn induces the production of pro-inflammatory cytokines and oxidative stress (12, 13). It is known that RAGE expression is low under physiological conditions and will increase its expression in an inflammatory environment (28), suggesting that RAGE levels are high in MS lesions. NF-κB activation, which plays a major role in MS pathology, is present in and around MS lesions, predominantly in the glial cells and infiltrated macrophages (29). Moreover, it has been suggested that there is a link between NF-κB related gene expression and clinical relapses (30, 31). Although AGEs in the CSF did not correlate with disease progression, we postulate that microglial and macrophage RAGE activation contribute, at least partly, to the increased NF-κB activation seen in MS lesions. This will in turn contribute to the inflammatory state of the microenvironment of the CNS.

Linear regression analysis showed no positive correlations between α-dicarbonyls and free AGEs with markers related to disease progression, and the same holds true for protein-bound AGEs. In fact, free MG-H1 in the CSF was negatively correlated with disease duration and protein-bound MG-H1 in the CSF was significantly negatively correlated with EDSS. One explanation for the decreased MG-H1 levels, free and protein-bound, with increasing disease progression and disability might be that patients in the progressive phase of the disease, which have most often a longer disease duration and score higher on the EDSS, experience less relapses and thus less inflammation (32). This could subsequently affect the MG-H1 levels in the CSF. An important factor to take into account is that free MG-H1 may be able to cross the blood-CSF barrier seen by the strong correlation between plasma and CSF free MG-H1. However, it is unclear whether free MG-H1 in the CSF origins from MS lesions and leaks into the periphery or whether free MG-H1 originates from the periphery and enters the CSF. This needs to be further investigated. It should also be mentioned that, although we corrected for medication use in our statistic model, most of these MS patients take anti-inflammatory treatments. These treatments inhibit the inflammatory component of the disease and may therefore interfere with the production of AGEs. Moreover, whether or not patients experience a relapse at the moment of CSF collection may have a significant impact on their AGE levels, as active inflammation is present during relapses, inducing AGE. Unfortunately, information whether patients experience a relapse at the time of sample collection is not available. Another explanation for the lack of correlation between α-dicarbonyl and AGE levels with disease progression markers of MS could be that α-dicarbonyls and AGEs reside in the CNS, intracellularly, and are not released into the CSF. Altogether, our findings indicate that use of AGE levels in the CSF as marker for disease progression is limited.

CSF α-dicarbonyls and free AGEs, but not protein-bound AGEs, correlate with their respective levels in the plasma. These results suggest that there is a lack of exchange of protein-bound AGEs between the plasma and CSF while α-dicarbonyls and free AGEs are easily exchanged between the plasma and CSF. CSF is produced in the CNS by the choroid plexus (33), which comprises the major part of the blood-CSF barrier and ensures separation of blood and CSF. The passage of solutes and nutrients is controlled by tight-junctions (34), and by various transporters (35). The crossing rate across the blood-CSF barrier is inversely correlated with the molecular weight of the substance, meaning the bigger the substance, the less crossing over the blood-CSF barrier occurs (36). This suggests that the small α-dicarbonyls and free AGEs, consisting of single modified amino acids, are able to pass the blood-CSF barrier more easily in contrast to the protein-bound AGEs. This may explain why we did not found correlations between protein-bound AGEs in the CSF and in the plasma.

In conclusion, we show that protein-bound MG-H1 is increased in MS lesions compared to white matter of NDCs and is present in activated GFAP^+^ astrocytes. This indicates that MGO-derived AGEs formed in glycolytic astrocytes may activate RAGE-positive microglia/macrophages in MS lesions and contribute to the inflammatory microenvironment. Further research is needed to elucidate whether lowering MG-H1 production in MS lesions is a therapeutic option for MS.

## Author Contributions

SW performed the experiments, analyzed the data, and wrote the manuscript. TV supervised the experiments and wrote the manuscript. JS designed methods and performed measurements. JvH supervised immunohistochemistry, analyzed data, and revised the manuscript. SA provided CSF samples and revised the manuscript. VS research design and biobank sample collection. CS supervised the experiments and revised the manuscript. JH supervised the experiments and revised the manuscript. KW supervised the experiments and wrote the manuscript.

### Conflict of Interest Statement

The authors declare that the research was conducted in the absence of any commercial or financial relationships that could be construed as a potential conflict of interest.

## Supplementary Material

Supplemental Figure S1 Post-mortem delay is not correlated with α-dicarbonyl, free, and protein-bound AGE levels. Using linear regression analysis, the correlation between post-mortem delay (PDM) and α-dicarbonyls MGO and GO **(A)**, protein-bound (PB) CML, CEL, and MG-H1 **(B)** and free CML, CEL, and MG-H1 **(C**), was determined. Data shows regression line with 95% confidence band.

Supplemental Figure S2 MGO-derived AGE accumulates predominantly in astrocytes in white matter of NDCs. Staining of MGO-derived AGE (red, TRITC) combined with GFAP (green, FITC) **(A)**, Iba1 (green, FITC) **(B)**, and neurofilament (NF) (green, FITC) **(C)** show that MGO-derived AGE accumulates in astrocytes in white matter of NDCs as indicated by the white arrows. Nuclei were stained with DAPI (blue). Representative of *n* = 4 staining.

## Abbreviations

3DG: 3-deoxyglucosone
AGEs: Advanced glycation endproducts
CEL: N^ε^-(1-carboxyethyl)lysine
CML: N^ε^-(carboxymethyl)lysine
CNS: Cental nervous system
CSF: Cerebrospinal fluid
EDSS: Expanded disability scale score
GLO1: Glyoxalase1
GLO2: Glyoxalase2
GO: Glyoxal
MG-H1: N^δ^-(5-hydro-5-methyl-4-imidazolon-2-yl)-ornithine
MGO: Methylglyoxal
MS: Multiple sclerosis
NDCs: Non-demented controls
RAGE: Receptor.

## References

[1] Ellwardt E Zipp F. Molecular mechanisms linking neuroinflammation and neurodegeneration in MS. Exp Neurol. (2014) 262:8–17. 10.1016/j.expneurol.2014.02.006 24530639

[2] Hemmer B Kerschensteiner M Korn T. Role of the innate and adaptive immune responses in the course of multiple sclerosis. Lancet Neurol. (2015) 14:406–19. 10.1016/S1474-4422(14)70305-9 25792099

[3] Bogie JF Stinissen P Hendriks JJ. Macrophage subsets and microglia in multiple sclerosis. Acta Neuropathol. (2014) 128:191–213. 10.1007/s00401-014-1310-2 24952885

[4] Nair A Frederick TJ Miller SD. Astrocytes in multiple sclerosis: a product of their environment. Cell Mol Life Sci. (2008) 65:2702–20. 10.1007/s00018-008-8059-5 18516496 PMC2858316

[5] Scalfari A Neuhaus A Daumer M Muraro PA Ebers GC. Onset of secondary progressive phase and long-term evolution of multiple sclerosis. J Neurol Neurosurg Psychiatry. (2014) 85:67–75. 10.1136/jnnp-2012-304333 23486991

[6] Itoh Y Esaki T Shimoji K Cook M Law MJ Kaufman E. Dichloroacetate effects on glucose and lactate oxidation by neurons and astroglia *in vitro* and on glucose utilization by brain *in vivo*. Proc Natl Acad Sci USA. (2003) 100:4879–84. 10.1073/pnas.0831078100 12668764 PMC153649

[7] Kelly B O'Neill LA. Metabolic reprogramming in macrophages and dendritic cells in innate immunity. Cell Res. (2015) 25:771–84. 10.1038/cr.2015.68 26045163 PMC4493277

[8] Orihuela R McPherson CA Harry GJ. Microglial M1/M2 polarization and metabolic states. Br J Pharmacol. (2016) 173:649–65. 10.1111/bph.13139 25800044 PMC4742299

[9] Lange JN Wood KD Knight J Assimos DG Holmes RP. Glyoxal formation and its role in endogenous oxalate synthesis. Adv Urol. (2012) 2012:819202. 10.1155/2012/819202 22567004 PMC3332067

[10] Allaman I Belanger M Magistretti PJ. Methylglyoxal, the dark side of glycolysis. Front Neurosci. (2015) 9:23. 10.3389/fnins.2015.00023 25709564 PMC4321437

[11] Vistoli G De Maddis D Cipak A Zarkovic N Carini M Aldini G. Advanced glycoxidation and lipoxidation end products (AGEs and ALEs): an overview of their mechanisms of formation. Free Radic Res. (2013) 47(Suppl. 1):3–27. 10.3109/10715762.2013.815348 23767955

[12] Singh R Barden A Mori T Beilin L. Advanced glycation end-products: a review. Diabetologia. (2001) 44:129–46. 10.1007/s001250051591 11270668

[13] Gaens KH Stehouwer CD Schalkwijk CG. Advanced glycation endproducts and its receptor for advanced glycation endproducts in obesity. Curr Opin Lipidol. (2013) 24:4–11. 10.1097/MOL.0b013e32835aea13 23298958

[14] Maessen DE Stehouwer CD Schalkwijk CG. The role of methylglyoxal and the glyoxalase system in diabetes and other age-related diseases. Clin Sci. (2015) 128:839–61. 10.1042/CS20140683 25818485

[15] Gaens KH Goossens GH Niessen PM van Greevenbroek MM van der Kallen CJ Niessen HW. Nepsilon-(carboxymethyl)lysine-receptor for advanced glycation end product axis is a key modulator of obesity-induced dysregulation of adipokine expression and insulin resistance. Arterioscler Thromb Vasc Biol. (2014) 34:1199–208. 10.1161/ATVBAHA.113.302281 24723555

[16] van Eupen MG Schram MT Colhoun HM Hanssen NM Niessen HW Tarnow L. The methylglyoxal-derived AGE tetrahydropyrimidine is increased in plasma of individuals with type 1 diabetes mellitus and in atherosclerotic lesions and is associated with sVCAM-1. Diabetologia. (2013) 56:1845–55. 10.1007/s00125-013-2919-8 23620061

[17] Hanssen NM Wouters K Huijberts MS Gijbels MJ Sluimer JC Scheijen JL. Higher levels of advanced glycation endproducts in human carotid atherosclerotic plaques are associated with a rupture-prone phenotype. Eur Heart J. (2014) 35:1137–46. 10.1093/eurheartj/eht402 24126878

[18] Stitt AW Li YM Gardiner TA Bucala R Archer DB Vlassara H. Advanced glycation end products (AGEs) co-localize with AGE receptors in the retinal vasculature of diabetic and of AGE-infused rats. Am J Pathol. (1997) 150:523–31. 9033268 PMC1858286

[19] Dalfo E Portero-Otin M Ayala V Martinez A Pamplona R Ferrer I. Evidence of oxidative stress in the neocortex in incidental Lewy body disease. J Neuropathol Exp Neurol. (2005) 64:816–30. 10.1097/01.jnen.0000179050.54522.5a 16141792

[20] Ahmed N Ahmed U Thornalley PJ Hager K Fleischer G Munch G. Protein glycation, oxidation and nitration adduct residues and free adducts of cerebrospinal fluid in Alzheimer's disease and link to cognitive impairment. J Neurochem. (2005) 92:255–63. 10.1111/j.1471-4159.2004.02864.x 15663474

[21] Sternberg Z Ostrow P Vaughan M Chichelli T Munschauer F. AGE-RAGE in multiple sclerosis brain. Immunol Invest. (2011) 40:197–205. 10.3109/08820139.2010.532267 21080832

[22] Sternberg Z Hennies C Sternberg D Wang P Kinkel P Hojnacki D. Diagnostic potential of plasma carboxymethyllysine and carboxyethyllysine in multiple sclerosis. J Neuroinflammation. (2010) 7:72. 10.1186/1742-2094-7-72 21034482 PMC2984414

[23] Wetzels S Wouters K Miyata T Scheijen J Hendriks JJA Schalkwijk CG Advanced glycation endproducts are increased in the animal model of multiple sclerosis but cannot be reduced by pyridoxamine treatment or glyoxalase 1 overexpression. Int J Mol Sci. (2018) 19:E1311 10.3390/ijms19051311 29702605 PMC5983766

[24] Hanssen NM Engelen L Ferreira I Scheijen JL Huijberts MS van Greevenbroek MM. Plasma levels of advanced glycation endproducts Nepsilon-(carboxymethyl)lysine, Nepsilon-(carboxyethyl)lysine, and pentosidine are not independently associated with cardiovascular disease in individuals with or without type 2 diabetes: the hoorn and CODAM studies. J Clin Endocrinol Metab. (2013) 98:E1369–73. 10.1210/jc.2013-1068 23780372

[25] Scheijen JL Schalkwijk CG. Quantification of glyoxal, methylglyoxal and 3-deoxyglucosone in blood and plasma by ultra performance liquid chromatography tandem mass spectrometry: evaluation of blood specimen. Clin Chem Lab Med. (2014) 52:85–91. 10.1515/cclm-2012-0878 23492564

[26] McLellan AC Phillips SA Thornalley PJ. The assay of S-D-lactoylglutathione in biological systems. Anal Biochem. (1993) 211:37–43. 10.1006/abio.1993.1229 8323036

[27] Barateiro A Afonso V Santos G Cerqueira JJ Brites D van Horssen J. S100B as a potential biomarker and therapeutic target in multiple sclerosis. Mol Neurobiol. (2016) 53:3976–91. 10.1007/s12035-015-9336-6 26184632

[28] Bierhaus A Nawroth PP. Multiple levels of regulation determine the role of the receptor for AGE (RAGE) as common soilin inflammation, immune responses and diabetes mellitus and its complications. Diabetologia. (2009) 52:2251–63. 10.1007/s00125-009-1458-9 19636529

[29] Mc Guire C Prinz M Beyaert R van Loo G. Nuclear factor kappa B (NF-kappaB) in multiple sclerosis pathology. Trends Mol Med. (2013) 19:604–13. 10.1016/j.molmed.2013.08.001 24007818

[30] Satoh J Misawa T Tabunoki H Yamamura T. Molecular network analysis of T-cell transcriptome suggests aberrant regulation of gene expression by NF-kappaB as a biomarker for relapse of multiple sclerosis. Dis Markers. (2008) 25:27–35. 10.1155/2008/824640 18776589 PMC3827813

[31] Lindsey JW Agarwal SK Tan FK. Gene expression changes in multiple sclerosis relapse suggest activation of T and non-T cells. Mol Med. (2011) 17:95–102. 10.2119/molmed.2010.00071 20882258 PMC3022990

[32] Compston A Coles A. Multiple sclerosis. Lancet. (2008) 372:1502–17. 10.1016/S0140-6736(08)61620-7 18970977

[33] Damkier HH Brown PD Praetorius J. Cerebrospinal fluid secretion by the choroid plexus. Physiol Rev. (2013) 93:1847–92. 10.1152/physrev.00004.2013 24137023

[34] Praetorius J Damkier HH. Transport across the choroid plexus epithelium. Am J Physiol Cell Physiol. (2017) 312:C673–86. 10.1152/ajpcell.00041.2017 28330845

[35] Marques F Sousa JC Brito MA Pahnke J Santos C Correia-Neves M. The choroid plexus in health and in disease: dialogues into and out of the brain. Neurobiol Dis. (2017) 107:32–40. 10.1016/j.nbd.2016.08.011 27546055

[36] Pardridge WM. CSF, blood-brain barrier, and brain drug delivery. Expert Opin Drug Deliv. (2016) 13:963–75. 10.1517/17425247.2016.1171315 27020469

