# Supplementary figures and images for: Methylglyoxal-Derived Advanced Glycation Endproducts Accumulate in Multiple Sclerosis Lesions
Source: Front Immunol. 2019 Apr 24;10:855. doi: 10.3389/fimmu.2019.00855 (PMC6491451; doi:10.3389/fimmu.2019.00855)

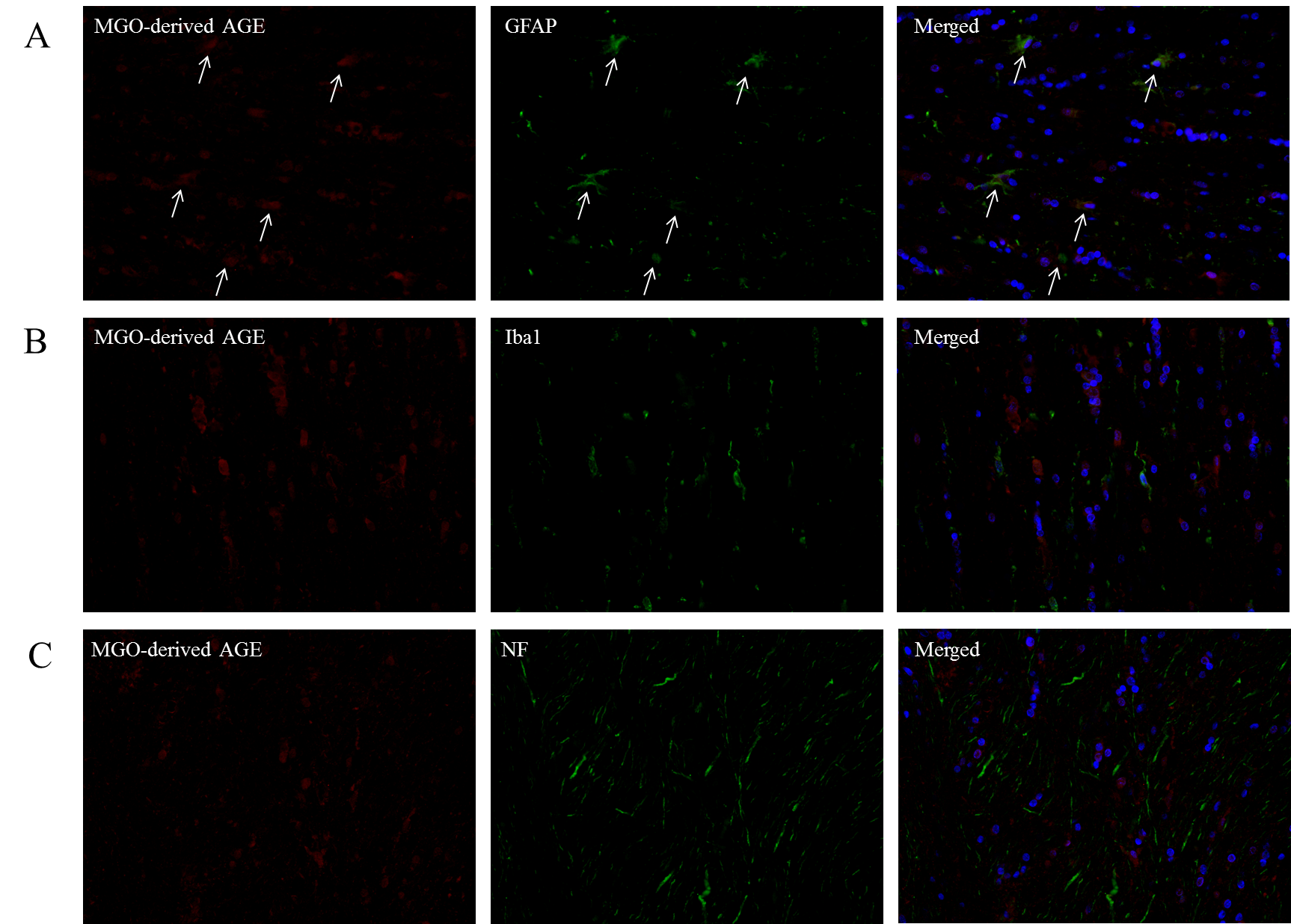

Supplement: Supplemental Figure S2 — MGO-derived AGE accumulates predominantly in astrocytes in white matter of NDCs. Staining of MGO-derived AGE (red, TRITC) combined with GFAP (green, FITC) (A), Iba1 (green, FITC) (B), and neurofilament (NF) (green, FITC) (C) show that MGO-derived AGE accumulates in astrocytes in white matter of NDCs as indicated by the white arrows. Nuclei were stained with DAPI (blue). Representative of n = 4 staining. [file Image_2.TIF]
